# Royal Medical Appointments for Scotland

**Published:** 1838-04

**Authors:** 


### ROYAL MEDICAL APPOINTMENTS FOR SCOTLAND.

*St. James's Palace; March* 3.

The Queen has been pleased to appoint the following Gentlemen upon her Majesty's Medical Establishment for Scotland:—Physicians in Ordinary: John Abercromby, M.D.; James Home, M.D.; John Thomson, M.D.; William P. Alison, M.D.—Surgeons in Ordinary: Sir George Ballingall, David M'Lagen, M.D.; James Syme, Esq.—Physicians Extraordinary: Andrew Combe, M.D.; Robert Spittal, M.D.—Surgeon Extraordinary: John Scott, M.D.—Surgeon Oculist: William Mackenzie, M.D. — Surgeon Dentists: Robert Nasmyth, Esq.; David Wemyss Jobson, Esq.

### *Additional Appointments for England.*

Surgeon Oculist: Henry Alexander, Esq.—Aurist Operator: William Maule, Esq.

[All the above appointments are not merely unexceptionable but excellent, and fully justify our observations at p. 584, respecting the character of the preceding nominations of the same kind. It has heretofore been, we fear, a somewhat rare event, in the bestowal of courtly honours, to see merit alone determining the selection of the individuals to be honoured. On the present occasion, as in the case of the Royal Medical Staff for England, we feel assured that the nominations will give general satisfaction to the profession; a circumstance, we will venture to assert, that has not before occurred since the accession of George the Third. If the rule now adopted is adhered to in future, the Royal Calendar will become a register of merit.]

---

